# Emergence of synaptic and cognitive impairment in a mature-onset APP mouse model of Alzheimer’s disease

**DOI:** 10.1186/s40478-019-0670-1

**Published:** 2019-02-22

**Authors:** Sarmi Sri, Chrysia-Maria Pegasiou, Chantal Abbigail Cave, Katie Hough, Natalie Wood, Diego Gomez-Nicola, Katrin Deinhardt, David Bannerman, V. Hugh Perry, Mariana Vargas-Caballero

**Affiliations:** 1 School of Biological Sciences and Institute for Life Sciences, University of Southampton, Southampton, SO17 1BJ UK; 2 Department of Experimental Psychology, University of Oxford, Oxford, OX1 3TA UK

## Abstract

The synaptic changes underlying the onset of cognitive impairment in Alzheimer’s disease (AD) are poorly understood. In contrast to the well documented inhibition of long-term potentiation (LTP) in CA3-CA1 synapses by acute Aβ application in adult neurons from rodents, young amyloid precursor protein (APP) transgenic mouse models often, surprisingly, show normal LTP. This suggests that there may be important differences between mature-onset and developmental-onset APP expression/ Aβ accumulation and the ensuing synaptic and behavioural phenotype. Here, in agreement with previous studies, we observed that developmental expression of APP_Sw,Ind_ (3–4 month old mice from line 102, PLoS Med 2:e355, 2005), resulted in reduced basal synaptic transmission in CA3-CA1 synapses, normal LTP, impaired spatial working memory, but normal spatial reference memory. To analyse early Aβ-mediated synaptic dysfunction and cognitive impairment in a more mature brain, we used controllable mature-onset APP_Sw,Ind_ expression in line 102 mice. Within 3 weeks of mature-onset APP_Sw,Ind_ expression and Aβ accumulation, we detected the first synaptic dysfunction: an impairment of LTP in hippocampal CA3-CA1 synapses. Cognitively, at this time point, we observed a deficit in short-term memory. A reduction in basal synaptic strength and deficit in long-term associative spatial memory were only evident following 12 weeks of APP_Sw,Ind_ expression. Importantly, the plasticity impairment observed after 3 weeks of mature-onset APP expression is reversible. Together, these findings demonstrate important differences between developmental and mature-onset APP expression. Further research targeted at this early stage of synaptic dysfunction could help identify mechanisms to treat cognitive impairment in mild cognitive impairment (MCI) and early AD.

## Introduction

Direct evidence from studies of the human brain suggests that hippocampal shrinkage [24] and synapse loss [18, 52] occur early in the pre-symptomatic and MCI phases of AD. Intervention at these early stages is becoming increasingly attractive from a therapeutic point of view as there is the potential to remove disease triggers and halt neurodegeneration prior to overt memory loss [28].

Human studies have provided a strong causal link between APP cleavage/Aβ production and the manifestation of AD [30]. Aβ can have potent synaptotoxic effects acutely or chronically in a wide variety of research models [7]. Acute Aβ application to neurons in culture or brain slices is sufficient to drive synaptic impairment within minutes to hours [14, 45, 55, 56, 66], and short-term exposure to Aβ in vivo can cause both synaptic and cognitive dysfunction in rodents within hours to days [11, 15, 41, 57].

Transgenic APP models allow for the analysis of chronic Aβ exposure and brain accumulation that could lead to a better understanding of the emergence and progression of cognitive impairment in AD. However, to date, animal research in AD has not led to a therapy and it is essential to continue the refinement of animal models to translate pre-clinical studies into relevant knowledge that can result in a disease modifying strategy. The vast majority of available AD mouse models express proteins with familial disease-causing mutations starting from embryonic or early postnatal development, and can thus be considered as developmental-onset models of AD. For example in the J20 line the PDGF promoter driven expression of APP_Sw,Ind_ starts at embryonic day 15 (E15) [51], in the Tg2576 line the PrP promoter driven expression of APP_Sw_ starts at E12 [1], and in the TASTPM line the Thy1 promoter driven expression of APP_Sw_ and Psen1 M146 V start at postnatal day 7 (P7) [12].

The use of these developmental-onset AD models raises a number of key issues. Firstly, intrinsic APP is developmentally expressed [21] and promotes synapse formation [68] and neuronal migration [72], and the further consequences of overexpressing mutant APP during development are still unclear. Secondly, differences in composition of glutamatergic synapses [36, 38] between developing and more mature mice can affect their responses to Aβ. For example LTP is affected by acute Aβ exposure in juvenile (P16–28), but not in postnatal (P8-P9) mouse hippocampal circuits [62]. Following developmental expression of Aβ in embryonic or postnatal animals, it is unknown whether compensatory effects make these circuits resilient to chronic Aβ exposure. Thus, overexpression of APP during development may cause complex and confounding effects on the observed phenotype. Thirdly, most behavioural tests cannot be performed in immature mice (e.g. younger than 6 weeks) and therefore it has not been possible to assess memory in young mice with developmental-onset of Aβ accumulation.

To investigate the emergence of both synaptic and cognitive impairments following Aβ accumulation in mice, we used the line 102 model, an inducible Tet-Off transgenic model that can be analyzed either as a developmental-onset AD model [35], or as an inducible AD model. Following previous work [23] we induced APP expression at 6 weeks of age –once key developmental processes have largely taken place; e.g. the peaks of neurogenesis and myelination rate have passed [20, 53]. Furthermore, post-natal changes in expression of synaptic proteins have largely stabilized [29] including GluN2A and GluN2B protein expression [36]. We thus refer to this model as mature-onset APP expression. Using electrophysiological, biochemical and behavioral analyses we characterized the emergence of cognitive and synaptic dysfunction in both developmental-onset and mature-onset versions of the line 102 model. Mapping the emergence and progression of deficits in synaptic function and cognition in this mouse model will help define the mechanisms underpinning memory loss as a consequence of rising levels of Aβ species and serve as a model for testing therapeutic strategies relevant to MCI and early AD.

## Materials and methods

### Animals

**Fig. 1:**
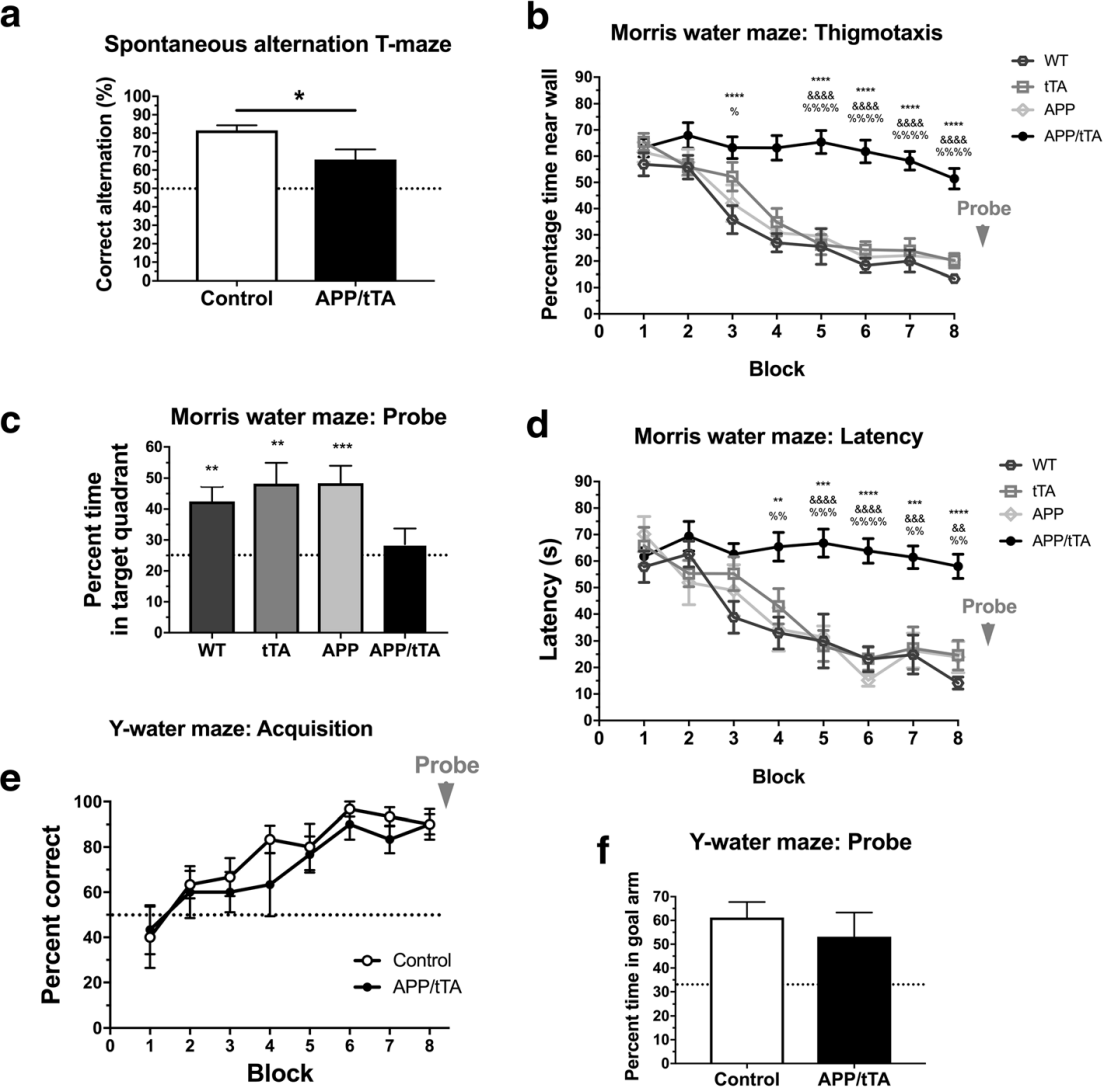
Developmental onset APP/tTA mice display impaired spatial working but normal spatial reference memory. **a** Developmental onset APP/tTA mice showed reduced spontaneous alternation in the T-maze (control mea*n* = 81.4 ± 2.8, *n* = 9; APP/tTA mea*n* = 65.8 ± 5.4, n = 9). Dashed line represents chance level of performance of 50%. **b** Developmental onset APP/tTA mice exhibited impaired performance on the Morris water maze, as evidenced by longer latency to platform scores compared to control WT, tTA and APP mice (WT *n* = 10, tTA n = 8, APP n = 9, APP/tTA *n* = 15). * WT vs. APP/tTA, ^%^ tTA vs. APP/tTA, ^&^ APP vs. APP/tTA. **c** Probe trial performed at the end of MWM testing showed a significant difference in time spent within the target quadrant. Control groups, but not APP/tTA, performed significantly better compared to chance performance of 25% (dashed line; WT mean = 42.5 ± 4.7; tTA mean = 48.2 ± 6.8; APP mean = 48.4 ± 5.65; APP/tTA mean = 28.6 ± 5.10). **d** Thigmotaxis analysis, measuring time spent within maze perimeter, showed that developmental onset APP/tTA mice were more thigmotaxic compared to control littermates. * WT vs. APP/tTA, ^%^ tTA vs. APP/tTA, ^&^ APP vs. APP/tTA. **e** Developmental onset APP/tTA mice showed normal spatial reference memory acquisition on the Y-water maze (control *n* = 6, APP/tTA n = 6). Dashed line represents chance level of performance of 50%. **f** Developmental onset APP/tTA mice were not significantly different from control littermates during probe trial testing (control mean = 58.8 ± 3.65; APP/tTA mean = 54.5 ± 8.97). Dashed line represents chance level of performance of 33.3%

Animal care and experimental procedures were conducted in accordance with UK Home Office regulations under the Animals (Scientific Procedures) Act of 1986. Mice were housed in groups of 2 to 6, under a 12/12 h light (6 am-6 pm)/dark(6:01 pm-5:59 am) cycle at 21 °C, with food and water *ad libitum*. All efforts were made to minimize experimental animal numbers. Transgenic APP and tTA mice from mouse line tetO-APPswe/ind (line 102) were generously provided by Joanna Jankowsky, Baylor School of Medicine. APP mice contained the chimeric mouse APP695 gene with a humanized Aβ domain containing the Swedish (APP KM670/671NL) and Indiana (APP V717F) mutations (APP_Sw,Ind_) driven by the tetO promoter while tTA mice contained the tetracycline transactivator (tTA) gene under the control of the CaMKIIα promoter. The colony was maintained by crossing single transgenic tTA and APP mice. The age of mice analyzed ranged from 6 to 35 weeks analysed with age-matched controls as indicated in each section. Following our observations of identical performance in the Morris water maze (Fig. 1b-d) for WT and single transgenic (APP or tTA) mice, these control genotypes were treated equally in all other tests. To control for age and sex effects, in each experiment littermate matched pairs were used and each matched pair consisted of a double transgenic APP/tTA mouse (expressing APP) and a single transgenic (control genotypes: WT, APP or tTA) of the same sex whenever possible (88% of our matched pairs were of the same sex), and overall we used 52% females, 48% males. The experimenter was blind to mouse genotype throughout all experimental procedures and data analysis.

### Doxycycline administration

Following [35], APP_Sw,Ind_ expression was suppressed by feeding doxycycline (dox) containing chow to pregnant females and females with litters, and later on weaning pups onto the dox diet. We used a high dose of dox (625 mg/kg, TestDiet Limited) given that we observed mild hyperactivity and weight loss in pilot experiments in double transgenics at 200 mg/kg possibly owing to enhanced transgene leakage (data not shown). At 6 weeks of age, the diet was switched to normal chow (RM-1, Special Diet Services, UK) to initiate APP_Sw,Ind_ expression and these animals are referred to as mature-onset mice. To achieve developmental-onset APP expression, breeding pairs and weaned mice were fed normal chow and mice were analyzed between 8 and 12 weeks of age.

### Acute brain slice preparation and electrophysiology

Following [56], mice were anaesthetised using isoflurane and decapitated. The brain was quickly extracted and submerged in artificial cerebrospinal fluid (aCSF) ~ 300 mOsm containing 126 mM NaCl, 2 mM CaCl_2_, 10 mM glucose, 2 mM MgSO_4_.7H_2_O, 3 mM KCl, 1.25 mM NaH_2_PO_4_.2H_2_O and 26.4 mM NaHCO_3_ bubbled with carbogen gas (95% O_2_, 5% CO_2_). Sagittal brain slices of 400 μm thickness were cut in ice-cold ACSF solution using a vibrating microtome (Campden Instruments, 7000 smz2). In older animals (29-weeks-off-dox group), 1 mM kynurenic acid was added to the ACSF during slicing to improve slice viability [22]. All slices were transferred to a submerged-type holding chamber and incubated in oxygenated ACSF at room temperature for approximately 60 min as a slice recovery period. Slices were then transferred to the recording interface chamber where they were continuously perfused with oxygenated ACSF at 30 °C with a flow rate of about 2 ml/min. Slices were allowed to recover in the interface chamber for a further 60 min before any recordings were made. Field excitatory postsynaptic potentials (fEPSPs) were recorded in the CA1 *stratum radiatum,* using glass microelectrodes filled with ACSF, in response to stimulation of the Schaffer collateral pathway using 50 μs pulses delivered via a stainless-steel electrode. Synaptic strength was assessed by generating input-output (I-O) curves assessing the initial slope of the rising phase of the average fEPSP (*N* = 3 repetitions) in response to stimulation strengths ranging from 20 to 200 μA. Higher stimulation amplitudes or repetitions were not used to avoid induction of plasticity changes. Stimulation was subsequently set to a value that elicited a fEPSP of ~ 40% of the maximum fEPSP amplitude to allow for enhancement of the response without spiking after LTP. Paired-pulse facilitation (PPF) was assessed with a pair of stimuli delivered at an inter-stimulus interval of 40 ms. The PPF ratio of the resulting average fEPSP waveform (N = 3 repetitions) was calculated by dividing the amplitude of the second fEPSP response by the amplitude of the first. Example traces for I-O curves and PPF are the average of 3 individual responses.

Baseline response for long term potentiation (LTP) was obtained by stimulating the Schaffer collateral pathway every 10 s and recording the fEPSP slope for a minimum of 30 min. Once a stable baseline was observed, LTP was induced using a theta burst stimulation paradigm (TBS) [47], made up of three trains separated by 15 s. Each train was composed of ten bursts at 5 Hz, each burst containing four pulses at 100 Hz. fEPSP slopes were normalised to the last 10 min of baseline average. LTP value was calculated by averaging fEPSPs 50–60 min after LTP induction. Example traces for LTP are the average of 6 individual responses.

The criterion for inclusion of I-O and PP data was the presence of an EPSP with a distinguishable volley upon stimulation of the Schaffer collaterals. If the EPSP was stable for the duration of the LTP baseline, then LTP was induced and measured. The number of LTP experiments to be performed was limited due to the overall time window of acute brain slice integrity (we recorded for ~ 8 h after dissection). Overall, for IO curves and PP data we used an average of 5.10 ± 1.93 slices per mouse, and for LTP an average of 2.63 ± 0.63 slices per mouse. Data from different slices from one animal were averaged and the average of those slices was counted as *N* = 1. All mice used for electrophysiology were experimentally naïve (i.e. they had not been used previously in the behavioural tasks described below).

### Behavioural assays

Behavioural assays were performed in developmental-onset or mature-onset mice. Developmental-onset mice were fed normal diet throughout their lifespan. Mature-onset mice were bred on doxycycline diet for 6 weeks and then switched to normal diet to “switch on” the APP_Sw,Ind_ transgene for 2, 3 or 12 weeks as indicated in each section below. Some tests were performed in mice maintained on the dox diet throughout to test for the potential impact of transgene leakage as indicated in each section. All tests were done in experimentally naïve mice.

### Spontaneous alternation in the T-maze

An enclosed T-maze of grey UPVC (30 cm long, 10 cm wide, 20 cm high) with sliding guillotine-type doors present at the arm entrances was used [17]. Each trial consisted of two consecutive runs; a trial run and a choice run. During the trial run the mouse was placed at the end of the start arm and allowed to enter either goal arm by choice, where it was then confined for 30 s by sliding down the door. Following this, the mouse was immediately removed from the maze. With both doors open, the mouse was placed in the start arm for the choice run. The alternation criterion was for the mouse to enter the opposite goal arm during the choice run (scoring 1 in such case, and 0 if it entered the same arm). Each mouse was tested with four trials per day with a minimum inter-trial interval of 30 min. 20 trials were performed in total over five consecutive days. The alternation score was the average score across the 20 trials. Different groups of mice were tested at 2, 3 and 12 weeks-off-dox. To test transgene leakage, 12-week-old mice that were bred on dox throughout their life were also tested.

### Morris water maze (MWM)

Following [2]**,** mice were trained in a water maze filled with opaque water maintained between 21 and 23 °C. We used two different mazes, one with a diameter of 2 m (Fig. 1b-d, Fig. 5c-e, Oxford) and one with a diameter of 1.2 m (Fig. 5a-b, Southampton); the platform with a 15 cm or 10 cm diameter, respectively, was kept submerged 1.5 cm below the water level. Trials were conducted over a two-week period. Acquisition training was performed during the first four days of each week and consisted of four trials a day with an inter-trial interval of 15 s. The mouse was placed facing the wall at one of the eight randomly allocated start locations and given 90 s to locate the hidden platform. If they failed to locate the platform within this time limit, they were guided to the submerged platform. Once on the platform, all mice spent 30 s on it before being removed from the water. A probe trial was performed at the end of week two. During the probe trial, the platform was removed and the mouse was given 60 s to swim freely in the maze. Thigmotaxis (defined here as an increased tendency to swim closer to the sidewall) was measured by assessing the time spent within 20 cm of the outer perimeter of the water maze (Figs. 1d and 5e). *Ethovision* video tracking system was used to acquire and analyse data. Developmental-onset mice were tested at 9–14 weeks. All other mice tested in the MWM were 8 weeks old, either bred on dox throughout to test transgene leakage or taken off the dox diet at 6 weeks of age to allow 2 weeks APP_Sw,Ind_ expression.

### Water escape Y-maze task (Y-water maze)

The apparatus consisted of an enclosed Y-maze made of clear Perspex with three arms (30 cm long, 8 cm wide, 20 cm high walls) separated by 120° [2]. The Y-water maze was filled with opaque water maintained between 21 and 23 °C and a platform with a diameter of 7 cm was kept submerged 1.5 cm below the water level. The platform was placed in a fixed location defined by the allocentric, extra-maze cues, at the end of one of the three arms (goal arm), which was counterbalanced across the different mouse groups. At the beginning of each trial, the mouse was placed at one of the two remaining arms (start arms). The order of start arm was varied pseudo-randomly across trials for each mouse (with no more than 2 consecutive starts from the same arm and equal numbers of starts from the arm to the left and right of the target arm in each block of trials) and was also counterbalanced across groups. The mouse was allowed to move freely around the maze until it reached the hidden platform. If the mouse did not reach the platform by 90 s it was guided to the platform. The mouse was allowed to remain on the platform for 30 s before being removed from the water maze. The first 9 days of testing involved acquisition training and consisted of five trials a day with an inter-trial interval of 15 s. For each trial, the first choice (first arm entered) was recorded and a score of 1 was given if the mouse entered the arm containing the platform first and a score of 0 given if it entered the other arm or re-entered the start arm. An arm entry was defined when a mouse placed all four paws into one of the goal arms. Performance for each individual animal was expressed as a percentage of correct first choices on each daily block of trials. On day 10, a probe trial was performed, where the platform was removed and the mouse was allowed to swim freely for 60 s. The percentage time spent in each arm was calculated during this probe trial. *Ethovision* video tracking system was used to acquire and analyse data. Mice were tested at 2, 3, and 12 weeks-off-dox.

### Tissue collection and homogenization

Mice from 6 to 18 weeks of age were terminally anesthetised with intraperitoneal injection of sodium pentobarbital and transcardially perfused with ice cold heparinised saline (0.9% saline containing 5000 U/L heparin). The brain was then carefully dissected and hemisected. The cortex and hippocampus were dissected and snap frozen on dry ice. Frozen hippocampal samples were lysed as previously described [23] and lysates were diluted at 2.5 mg/ml.

### Western blotting

15 μg of the resulting homogenate from above was separated on a 10% SDS-PAGE gel and transferred to nitrocellulose membrane. Membranes were blocked in TBS plus 0.1% Tween-20 and 5% dried skimmed milk for one hour. Membranes were probed overnight at 4 °C in the APP antibody 6E10 (1:11000; SIG-39320, BioLegend/ Covance) and GAPDH (1:11000; 5174, Cell Signalling) in 5% BSA and TBS plus 0.1% Tween-20. Membranes were then washed and probed with fluorescent secondary antibody, goat anti-mouse 800 (1:10,000, LICOR:926–32,210) and/or goat anti-rabbit-680 (1:10,000, LICOR:926–68,021), in 5% dried skimmed milk and TBS plus 0.1% Tween-20 and imaged using Odyssey CLx Infrared Imaging system (LICOR).

### Amyloid beta measurement

The Multispot ELISA (Mesoscale Discovery, Rockville, MD, V-PLEX Aβ Peptide Panel (6E10) Kit) was used to determine the concentration of Aβ peptide in mouse brain lysates. To maintain SDS concentrations at ≤0.1% SDS as per manufacturer’s instructions, the 2.5 mg/ml lysates prepared above were diluted 1:20 using Diluent 35 supplied by the manufacturer.

### Data analysis

Statistical significance was assessed with one-way, two-way or repeated measures (RM) ANOVA as indicated, followed by *post-hoc* analysis with Sidak’s correction except for Fig. 1 (APP and Aβ levels) where Fisher least significant difference was employed. In all statistical analyses, results N equals the number of mice (i.e. data from different slices from the same animal were averaged and counted as *N* = 1). All data are presented as mean ± SEM. Student’s t-tests were two-tailed and unpaired. Significant pairwise comparisons are highlighted within figures as follows: * *p* < 0.05, ** *p* < 0.01, *** *p* < 0.001, **** *p* < 0.0001.

## Results

### Developmental-onset of expression of APP_Sw,Ind_ in line 102 mice results in cognitive impairment, diminished basal synaptic function but normal CA3-CA1 LTP

First, to assess the phenotype of developmental-onset line 102, we analyzed mice bred in the absence of dox, so that APP expression driven by the CaMKIIα promoter commenced around P5 [6]. We tested spatial working memory using spontaneous alternation in the T-maze and found a significant impairment in APP/tTA mice which produce APP following Tet-Off activation of the promoter by tTA in the absence of dox (Fig. 1a; *t-*test: t_(16)_ = 2.58, *p* = 0.02). To assess spatial reference memory, we first used the Morris water maze. We analyzed all four genotypes of this transgenic mouse line: the three control genotypes: WT, tTA, APP and the affected genotype APP/tTA, and observed a substantial impairment in latency during acquisition in the double transgenic APP/tTA mice (Fig. 1b; RM ANOVA: genotype F_(3,40)_ = 20.09, *p* < 10^− 4^; training block F_(7,280)_ = 23.24, p < 10^− 4^; genotype x training block F_(21,280)_ = 3.31, *p* < 10^− 4^). A similar pattern of results was observed for path lengths (data not shown; RM ANOVA: genotype F_(3,40)_ = 15.5, p < 10^− 4^; training block F_(7,280)_ = 17.96, *p* < 10^− 4^; genotype x training block F_(21,280)_ = 2.84, p < 10^− 4^).

We also observed a significant effect of genotype in the time spent in the platform quadrant during the probe trial (Fig. 1c; one-way ANOVA: F_(3,40)_ = 3.16, *p* = 0.035), with APP/tTA mice performing close to chance levels of 25% (*t-*test: t_(22)_ = 0.55, *p* = 0.59), while the three control groups performed significantly better than chance (*t-*test: WT t_(17)_ = 3.50, *p* = 0.0028; tTA t_(17)_ = 3.24, *p* = 0.0048; APP t_(16)_ = 4.14, *p* = 0.0008).

However, closer inspection and analysis of these data revealed pronounced thigmotaxis in APP/tTA mice throughout the MWM testing (Fig. 1d; RM ANOVA: genotype F_(3,40)_ = 30.29, p < 10^− 4^; training block F_(7,280)_ = 54.26, p < 10^− 4^; genotype x training block F_(21,280)_ = 5.07, *p* < 10^− 4^), which followed a highly similar pattern to latency scores. Thus, although the impaired performance in the MWM is similar to that reported in many other APP models [48, 69], it is not possible to ascertain in our mice whether there is a genuine learning deficit is present in this behavioural paradigm. Indeed it is unclear whether the thigmotaxic behaviour that we observed in the MWM is a cause or a consequence of the spatial learning deficit in the developmental-onset 102 model. It is possible that the increase in thigmotaxis reflects a lack of engagement with this behavioural task (i.e. searching for the hidden platform) that could signal a phenotype unrelated to spatial memory, but which nevertheless appears like a spatial learning deficit. Alternatively, the increase in thigmotaxic behaviour in the developmental-onset mice could reflect a default strategy given an inability to learn about the spatial location of the platform.

To resolve this issue, we used an alternative water maze task in which differences in thigmotaxis would not confound the results. We used a hippocampal-dependent, swim-escape version of the Y-maze task (Y-water maze) which we have shown previously is robustly impaired by hippocampal lesions [43] and hippocampal GluN1 subunit ablation [2]. Mice were trained to locate a hidden escape platform which was situated consistently at the end of one of the goal arms, as defined by the extra-maze spatial cues. We assessed whether the mouse’s first choice was to enter the arm containing the escape platform (correct choice) and expressed this measure as the percentage of correct choices per block (see Methods). After the final block of training, we measured retention of memory by conducting a probe trial during which the platform was removed from the maze and the time spent in each of the maze arms was quantified. Surprisingly, spatial reference memory in the Y-water maze was comparable between APP/tTA mice and control littermates both during acquisition (Fig. 1e; RM ANOVA: genotype F_(1,10)_ = 0.65, *p* = 0.44; training block F_(7,70)_ = 10.89, *p* < 10^− 4^; genotype x training block F_(7,70)_ = 0.44, *p* = 0.87) and the probe trial (Fig. 1f; *t-*test: t_(10)_ = 0.52, *p* = 0.52). Thus, using a task that is much less likely to be confounded by thigmotaxis, we show that developmental-onset 8–12 week-old APP/tTA exhibit normal associative spatial reference (long-term) memory in contrast to their impaired spatial working (short-term) memory.

**Fig. 2:**
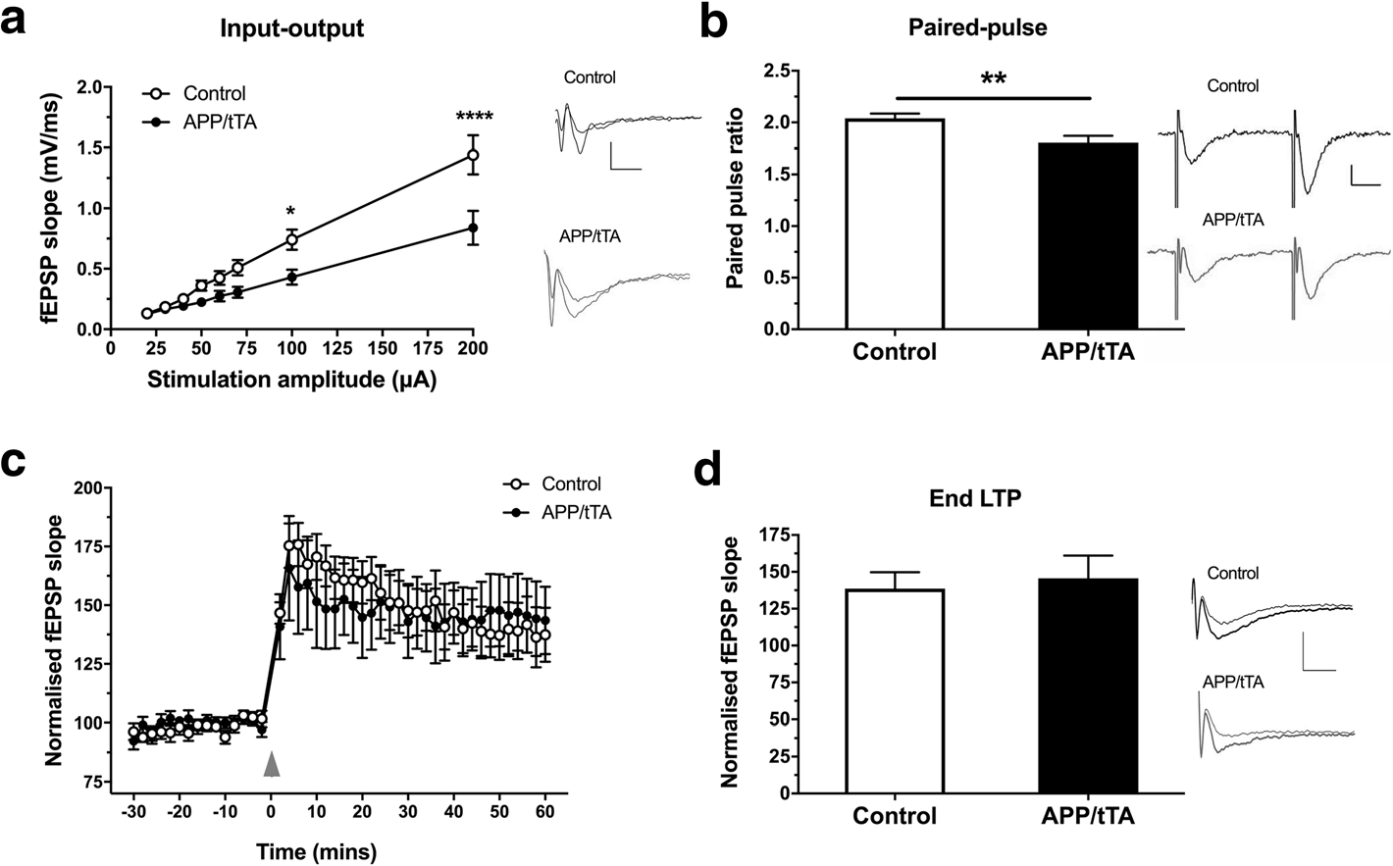
Developmental onset APP/tTA mice display impaired basal synaptic transmission but normal long term potentiation. **a** Input-output response was reduced in the CA3-CA1 pathway of developmental onset APP/tTA mice compared to control littermates (control n = 15, APP/tTA n = 15). Scale bar calibration: 5 ms, 0.5 mV. **b** Reduced paired-pulse response in developmental onset APP/tTA mice (control mean = 2 ± 0.05, *n* = 14; APP/tTA mean 1.8 ± 0.07, n = 14). Scale bar calibration: 10 ms, 0.2 mV. **c** TBS-induced LTP (arrowhead) was similar between developmental onset control and APP/tTA mice. **d** End LTP value, averaged 50–60 min after TBS, showed that APP/tTA mice exhibit a similar level of LTP to control littermates (control mea*n* = 138.6 ± 11.2, *n* = 12; APP/tTA mean 145.6 ± 15.4, n = 12). fEPSP example traces shown for time points immediately before (thin line) and 60 min after (thick line) LTP induction. Scale bar calibration: 5 ms, 0.5 mV

Next, to analyze hippocampal synaptic function in developmental-onset APP/tTA mice, we first obtained I-O curves at CA3-CA1 synapses in hippocampal slices and observed a significant impairment in APP/tTA mice compared to control littermates (Fig. 2a; RM ANOVA: genotype F_(1,28)_ = 7.35, *p* = 0.011; stimulation F_(7,196)_ = 80.98, *p* < 10^− 4^; genotype x stimulation F_(7,196)_ = 7.24, *p* < 10^− 4^), similar to that observed in other age-matched APP models [31, 33]. We also found reduced paired pulse responses in APP expressing mice (Fig. 2b; *t-*test: t_(26)_ = 2.86, *p* = 0.0083), consistent with previous observations in young TASTPM mice [16].

Interestingly, however, similar levels of TBS-induced LTP responses were observed between developmental-onset APP/tTA and control mice (Fig. 2c-d; *t-*test: t_(22)_ = 0.37, *p* = 0.72). These results are in agreement with previous observations showing normal CA3-CA1 LTP in young adult APP transgenics including APP/PS1 [26, 27], J20 [42], and Tg2576 mice [13, 19]. Thus, the behavioural and CA3-CA1 synaptic phenotype of developmental-onset line 102 APP_Sw,Ind_ mice is highly similar to that previously observed in other young APP transgenic models that also express APP from embryonic and/or postnatal development.

### Rapid increase of APP expression and Aβ levels in mature-onset APP/tTA mice

**Fig. 3:**
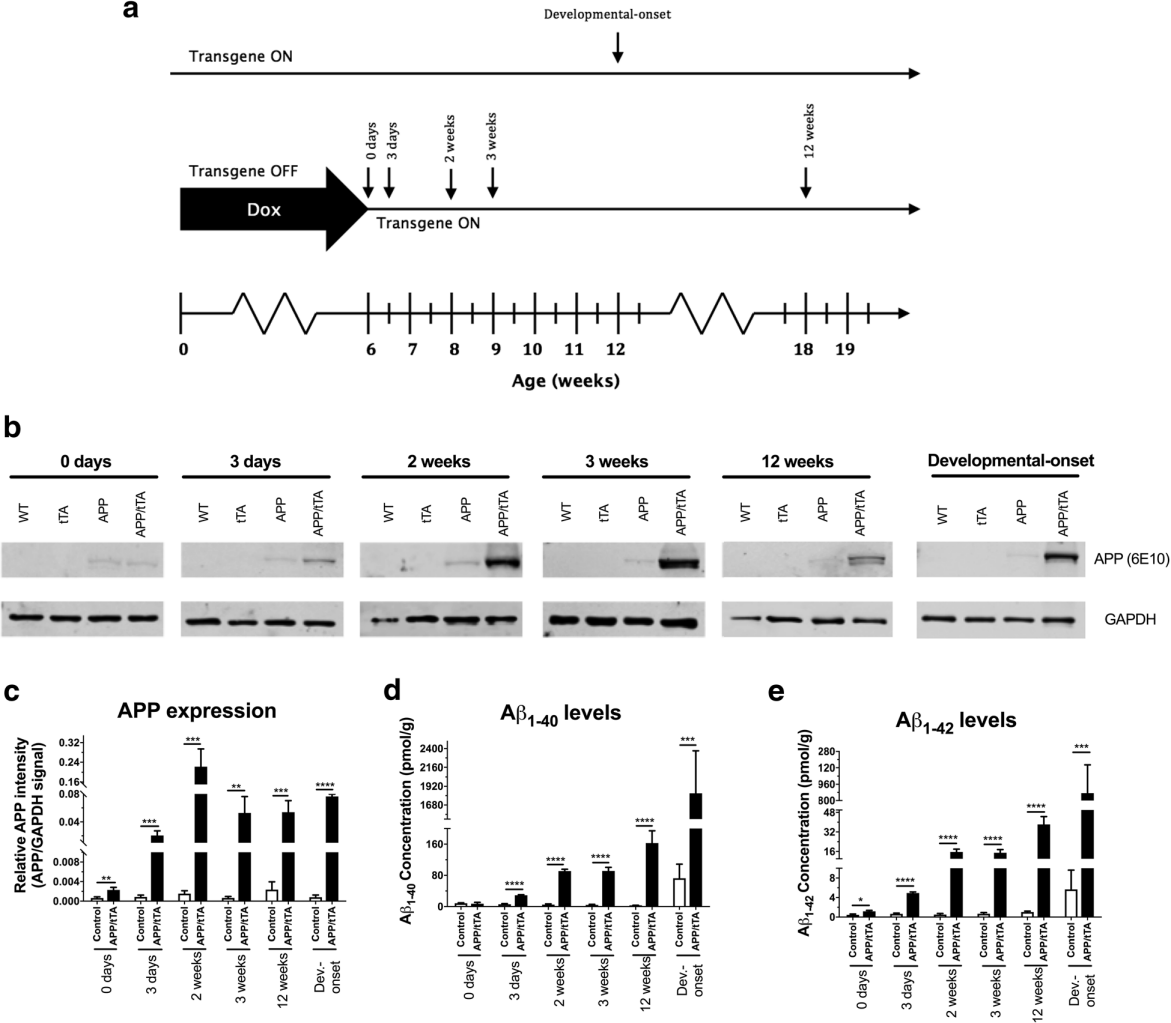
Induction of APP expression and increase in Aβ following dox removal in mature mice. **a** Experimental timeline showing time points at which APP expression and Aβ levels were measured. Hippocampal lysates were obtained from 12 week-old mice expressing the APP_Sw,Ind_ transgene from postnatal development (developmental onset) and from mature-onset mice reared on dox to suppress APP_Sw,Ind_ expression throughout life (0 days) and in mice 3 days, 2 weeks, 3 weeks and 12 weeks after dox removal. **b** Representative Western blots probing for APP expression (6E10) in WT, APP, tTA and APP/tTA mice at the different time points tested. GAPDH was used as a loading control. **c** Normalised APP signal intensity showed a significant effect of time of dox removal (control genotypes *n* = 9 for all time points, APP/tTA genotype *n* = 3 for all time points). **d** A rapid increase in Aβ_40_ was observed in APP/tTA mice following dox removal (control n = 9, APP/tTA n = 3 for all time points except 12 weeks where APP/tTA *n* = 7). **e** A rapid increase in Aβ_42_ was observed in APP/tTA mice following dox removal (control n = 9, APP/tTA n = 3 for all time points except 12 weeks where APP/tTA n = 7)

To analyze the impact of mature-onset APP expression in line 102 mice, we raised mice on a dox diet until 6 weeks of age (referred to as time 0). Then, mice were switched to a normal chow for either 3 days, 2 weeks, 3 weeks or 12 weeks (referred to as time-off-dox, Fig. 3a). We used Western blots to measure hippocampal APP expression for each of the four genotypes (Fig. 3b). As expected, we observed a rapid and significant increase in APP expression in APP/tTA mice following dox withdrawal (Fig. 3b-c; two way ANOVA: genotype F_(1,50)_ = 71.98, *p* < 10^− 4^; time-off-dox F_(4,50)_ = 23.26, *p* < 10^− 4^; genotype x time-off-dox F_(4,50)_ = 22.98, p < 10^− 4^). Post-hoc tests showed significantly higher levels of APP expression for APP/tTA mice at all tested time points, which peaked after 2 weeks-off-dox (Fig. 3b-c). A faint APP transgene “leakage” band was observed in APP/tTA mice that remained on dox (0 days). This band was, however, significantly enhanced after APP/tTA mice were taken off the dox diet for 3 days (Fig. 3b-c; *p* = 0.004). In addition, an APP band was also observed in single transgenic APP mice, in the absence of the tTA transgene, and did not change across the different time points tested (Fig. 3b). Importantly, single transgenic APP mice performed as well as WT and tTA controls in the MWM (Fig. 1b-d). This is consistent with previous work with tTA-driven mouse models where minimal transgene expression (“leakiness”) has been observed in single transgenic APP mice [31] with no effect on the phenotype.

We next examined whether there were APP expression differences between the mature-onset mice and developmental-onset APP mice described in the previous section. We compared APP levels in 12 weeks-off-dox mice (mature-onset, 18 weeks of age), against the 12-week-old developmental-onset mice (Fig. 3a), thus matching for length of expression (Fig. 3b-c). There was no significant difference in the APP expression levels between APP/tTA developmental-onset vs mature-onset APP/tTA mice (*t-*test: t_(4)_ = 1.3, *p* = 0.25).

In order to assess Aβ levels we used an immunoassay (Mesoscale platform) (Fig. 3d-e) and found a significant increase in both Aβ_40_ (Fig. 3d; two way ANOVA: genotype F_(1,54)_ = 77.8, *p* < 10^− 4^; time-off-dox F_(4,54)_ = 12.98, p < 10^− 4^; genotype x time-off-dox F_(4,54)_ = 14.92, *p* < 10^− 4^) and Aβ_42_ (Fig. 3e; two way ANOVA: genotype F_(1,54)_ = 76.01, *p* < 10^− 4^; time off dox F_(4,54)_ = 20.65, p < 10^− 4^; genotype x time-off-dox F_(4,54)_ = 19.5, p < 10^− 4^) in APP/tTA mice following dox withdrawal. Post-hoc tests showed that Aβ_40_ levels in APP/tTA mice at time 0 were comparable to controls (Fig. 3d; *t-*test: t_(10)_ = 0.42, *p* = 0.68), whereas Aβ_42_ levels were approximately three-fold higher than baseline levels (Fig. 3e; *t-*test: t_(10)_ = 2.3, *p* = 0.044) which is consistent with the APP leakage described above. A steep increase was observed thereafter in APP/tTA mice from 3 days after dox removal for both Aβ_40_ and Aβ_42_ (Fig. 3d-e; *p* < 10^− 4^ for all time points after dox removal). Thus, we show that removal of dox from the diet resulted in a rapid and highly significant increase in both APP expression and Aβ levels.

A comparison of Aβ levels between developmental-onset APP/tTA mice and time-of-expression matched mature-onset 12 weeks-off-dox mice showed a surprising seven-fold increase in Aβ_40_ levels and a twenty-fold increase in Aβ_42_ levels in developmental-onset APP/tTA mice compared to expression-matched mature-onset APP/tTA mice (Aβ_40_: Fig. 3d; two way ANOVA: genotype F_(1,24)_ = 66.97, *p* < 10^− 4^; time of expression onset F_(1,24)_ = 54.95, p < 10^− 4^; genotype x time of expression onset F_(1,24)_ = 46.42, p < 10^− 4^, Aβ_42_: Fig. 3e; two way ANOVA: genotype F_(1,24)_ = 61.37, *p* < 10^− 4^; time of expression onset F_(1,24)_ = 52.85, p < 10^− 4^; genotype x time of expression onset F_(1,24)_ = 51.69, p < 10^− 4^). This suggests stronger concentration-dependent Aβ effects in developmental-onset line 102 mice.

### Spatial working memory deficits in mature-onset APP/TTA mice following 3 weeks of APP_Sw,Ind_ expression

**Fig. 4:**
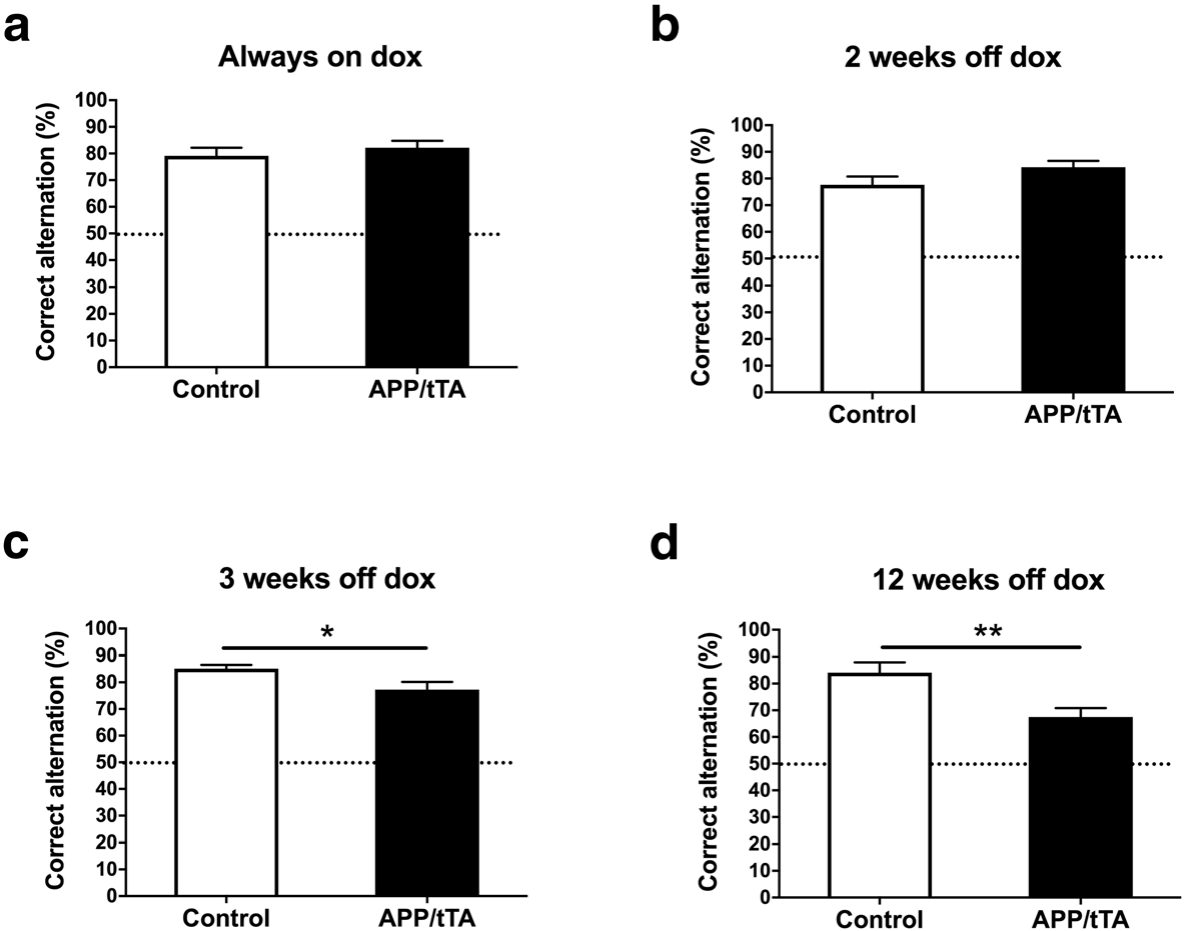
Emergence of spatial working memory deficit on the T-maze. **a** APP/tTA mice reared on dox to suppress APP_Sw,Ind_ throughout life performed similar to control littermates (control mean = 79.1 ± 3.07, *n* = 17; APP/tTA mea*n* = 82.2 ± 2.57, n = 17). Dashed line represents chance level performance of 50%. **b** Spontaneous alternation score was similar between APP/tTA mice expressing APP_Sw,Ind_ for 2 weeks (2 weeks-off-dox) and control littermates (control mean = 77.7 ± 3.06, *n* = 16; APP/tTA mean = 84.3 ± 2.34, n = 12). **c** A reduced spontaneous alternation score was observed in APP/tTA mice expressing APP_Sw,Ind_ for 3 weeks (3-weeks-off-dox; control mean = 85.1 ± 1.44, n = 14; APP/tTA mean = 77.3 ± 2.89, n = 14). **d** APP/tTA mice expressing APP_Sw,Ind_ for 12 weeks (12 weeks-off-dox) exhibited reduced alternation scores compared to control littermates (control mean = 84.1 ± 3.83, n = 8; APP/tTA mea*n* = 67.5 ± 3.38, *n* = 11)

We next investigated the effects of inducible, mature-onset, APP expression and Aβ accumulation on hippocampus dependent spatial working memory using spontaneous alternation behavior in the T-maze [17]. We observed a significant interaction between genotype and duration of APP_Sw,Ind_ expression (two-way ANOVA: F_(3,101)_ = 5.82, *p* = 0.001). We found that APP/tTA mice born and raised on dox since conception (always-on-dox), had alternation scores comparable to those of littermate controls (Fig. 4a; *t-*test: t_(32)_ = 0.78, *p* = 0.44). Similarly, APP/tTA mice taken off the dox diet for 2 weeks performed equally as well as control littermates (Fig. 4b; *t-*test: t_(26)_ = 1.61, *p* = 0.12). In contrast, APP/tTA mice taken off dox for 3 weeks had significantly lower spontaneous alternation scores compared to control littermates (Fig. 4c; *t-*test: t_(26)_ = 2.42, *p* = 0.023). A further reduction in spontaneous alternation scores was observed in APP/tTA mice that were taken off the dox diet for 12 weeks (Fig. 4d; *t-*test: t_(17)_ = 3.24, *p* = 0.0048). Thus, our results show that 3 weeks of APP_Sw,Ind_ expression, but not 2 weeks, is sufficient to drive a progressive deficit in hippocampal dependent spatial working memory in the T-maze.

### Mature-onset APP/tTA mice exhibit thigmotaxic behavior in the Morris water maze at 2 weeks-off-dox

**Fig. 5:**
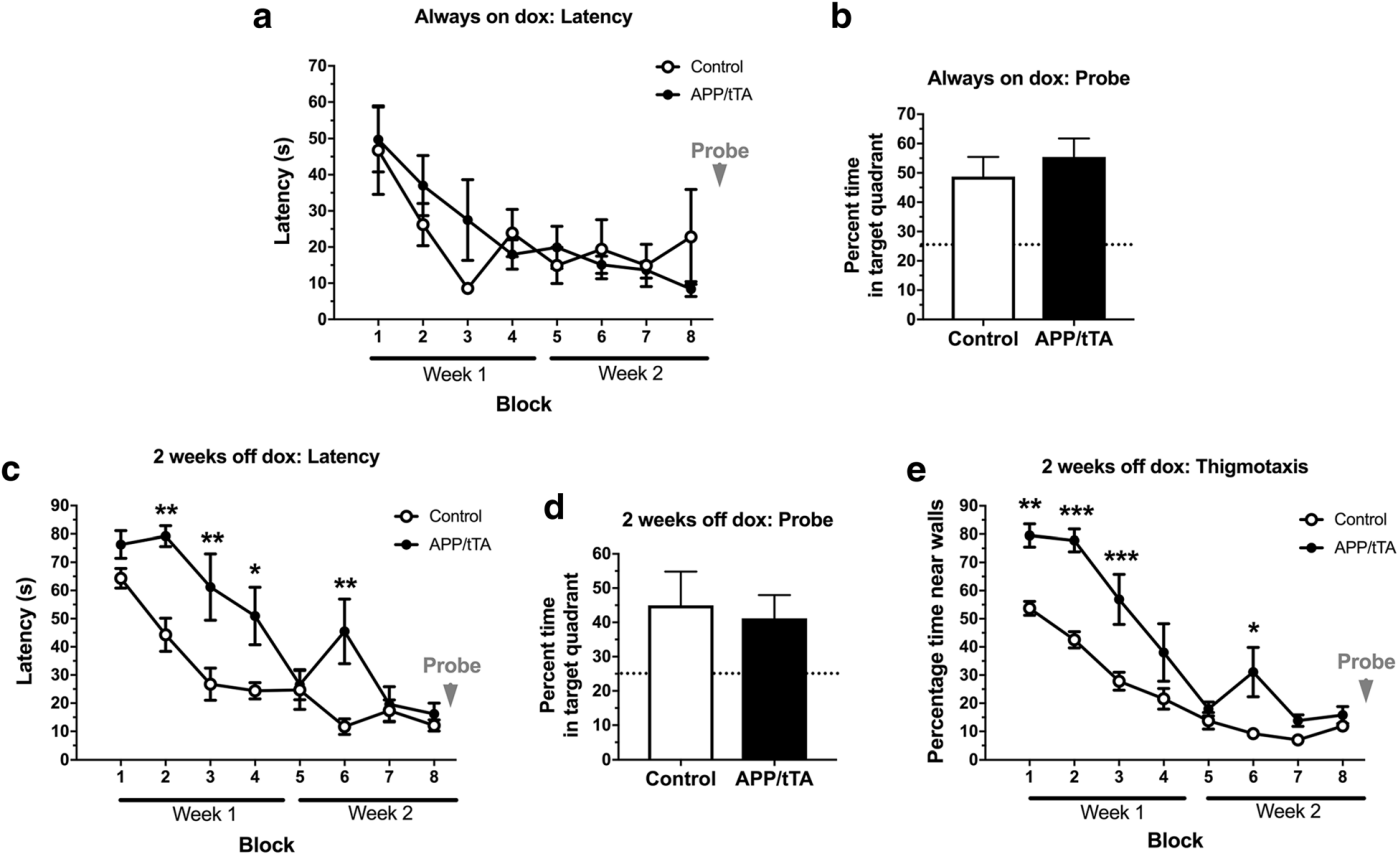
Mature-onset APP/tTA mice exhibit thigmotaxic behavior on the Morris water maze. **a** Analysis of latency to platform showed that APP/tTA mice reared on dox (always on dox) performed similar to control littermates (control n = 6, APP/tTA n = 6). **b** Probe trial performed at the end of water maze testing showed no significant difference in time spent within the target quadrant (control mean = 48.7 ± 6.72; APP/tTA mean = 55.4 ± 6.32). Dashed line represents chance level performance of 25%. **c** APP/tTA mice expressing APP_Sw,Ind_ for 2 weeks (2 weeks-off-dox) were slower to reach the platform compared to control littermates (control n = 6, APP/tTA n = 6). **d** Probe trial showed that APP/tTA mice expressing APP_Sw,Ind_ for 2 weeks spent a similar percentage of time in the platform quadrat as control littermates (control mean = 45 ± 9.82; APP/tTA mean = 41.2 ± 6.79). **e** Thigmotaxis analysis showed that APP/tTA mice expressing APP_Sw,Ind_ for 2 weeks were more thigmotaxic compared to control littermates

Next, we assessed MWM performance in the mature-onset line 102 model. First, we assessed the effects of baseline APP transgene leakage by studying 8-week-old mice kept always-on-dox. We found that these APP/tTA mice performed similarly to controls during acquisition (Fig. 5a; RM ANOVA: genotype F_(1,10)_ = 0.05, *p* = 0.82; training block F_(7,70)_ = 7.20, *p* < 10^− 4^; genotype x training block F_(7,70)_ = 1.45, *p* = 0.2) and the probe trial (Fig. 5b; *t-*test: t_(10)_ = 0.73, *p* = 0.48). These results showed that baseline performance in the MWM was similar for control and APP/tTA animals born and raised on the dox diet.

To analyze early effects of APP_Sw,Ind_ expression we next assessed MWM performance in mice taken off the dox diet at 6 weeks of age for 2 weeks (Fig. 5c-e). Analysis of latency to platform during training showed that APP/tTA mice took longer to reach the hidden platform compared to controls (Fig. 5c; RM ANOVA: genotype F_(1,10)_ = 13.28, *p* = 0.0045; training block F_(7,70)_ = 25.44, *p* < 10^− 4^; genotype x training block F_(7,70)_ = 3.63, *p* = 0.0021), and a similar pattern of results was observed for path lengths (data not shown; RM ANOVA: genotype F_(1,10)_ = 9.43, *p* = 0.05; training block F_(7,70)_ = 15.90, p < 10^− 4^; genotype x training block F_(7,70)_ = 3.41, *p* = 0.01).

Notably, on the very first trial we also observed a highly significant effect of genotype on thigmotaxic behaviour (Fig. 5e; F_(1,10)_ = 19.62, *p* = 0.0013), indicating basal differences prior to learning. Thigmotaxic behavior decreased with training and closely paralleled reductions in latency scores. By the end of training, both groups were escaping rapidly from the water with equivalent latencies and after the final training block both APP/tTA mice and control littermates spent a similar amount of time in the platform quadrant during the probe trial (Fig. 5d; *t-*test: t_(10)_ = 0.32, *p* = 0.76). Our results show that mature-onset APP/tTA mice that expressed APP for 2 weeks show increased thigmotaxic behavior in the MWM, they were able to learn the platform location and achieve a similar level of performance to controls by the end of training, as previously observed in mature-onset line 102 mice [23]. We cannot, however ascertain whether the observed differences in MWM performance during the early stages of training in this experiment are due to a learning deficit on this task given the confounding factors of thigmotaxis.

### A progressive decline in spatial reference memory in mature-onset APP/tTA mice after dox withdrawal

**Fig. 6:**
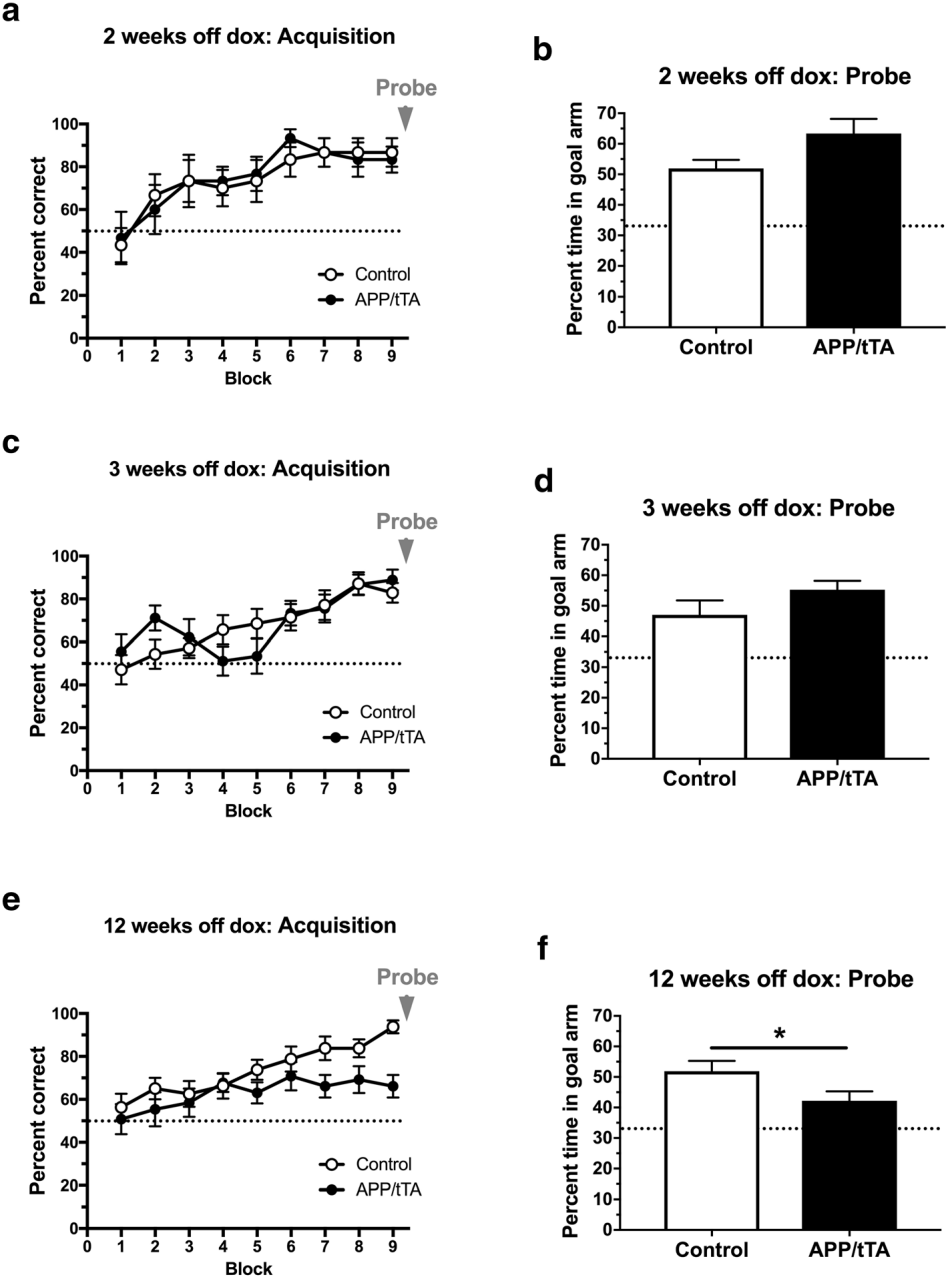
Emergence of spatial reference memory deficit on the Y-water maze. **a** APP/tTA mice expressing APP_Sw,Ind_ for 2 weeks (2 weeks-off-dox) performed similarly to control littermates (control n = 6, APP/tTA n = 6). Dashed line represents chance level of performance of 50%. **b** Probe trial performed at the end of water maze training showed APP/tTA mice performed slightly, but not significantly, better than control littermates (control mean = 51.9 ± 2.81; APP/tTA mean = 63.4 ± 4.75). Dashed line represents chance level of performance of 33.3%. **c** Analysis of acquisition showed that APP/tTA mice expressing APP_Sw,Ind_ for 3 weeks (3 weeks-off-dox) performed similarly to control littermates (control n = 14, APP/tTA n = 9). **d** Probe trial showed that APP/tTA mice performed similar to control littermates (control mean = 47 ± 4.74; APP/tTA mean = 55.3 ± 2.87). **e** RM-ANOVA analysis of acquisition showed that APP/tTA mice expressing APP_Sw,Ind_ for 12 weeks (12 weeks-off-dox) showed an effect of genotype but no interaction between genotype and block (control n = 16, APP/tTA n = 13). **f** Probe trial results showed that APP/tTA mice expressing APP_Sw,Ind_ for 12 weeks spent a significantly lower percentage time within the goal arm compared to control littermates (control mean = 51.9 ± 3.38; APP/tTA mean = 42.2 ± 3.10)

Given this potentially confounding thigmotaxis phenotype we observed in the MWM, we also assessed spatial reference memory using the Y-water maze. We found that mature-onset APP/tTA mice, expressing APP_Sw,Ind_ for 2 weeks, performed similarly to littermate controls during both acquisition training (Fig. 6a; RM ANOVA: genotype F_(1,10)_ = 0.011, *p* = 0.92; training block F_(8,80)_ = 7.08, *p* < 10^− 4^; genotype x training block F_(8,80)_ = 0.22, *p* = 0.99) and the probe trial (Fig. 6b; *t-*test: t_(10)_ = 2.08, *p* = 0.065). Similar results were obtained for mature-onset mice expressing APP_Sw,Ind_ for 3 weeks-off-dox (Fig. 6c-d; acquisition RM ANOVA: genotype F_(1,21)_ = 0.013, *p* = 0.91; training block F_(8,168)_ = 11.21, p < 10^− 4^; genotype x training block F_(8,168)_ = 1.89, p = 0.065; probe *t-*test: t_(21)_ = 1.29, *p* = 0.21). In contrast, we observed a significant effect of genotype in the acquisition phase for mature-onset APP/tTA mice at 12 weeks-off-dox (Fig. 6f; RM ANOVA: genotype F_(1,27)_ = 4.44, *p* = 0.045; training block F_(8,216)_ = 7.21, p < 10^− 4^; genotype x training block F_(8,216)_ = 1.63, *p* = 0.12). Furthermore, during the probe trial we found that mature-onset APP/tTA mice that expressed APP_Sw,Ind_ for 12 weeks spent less time in the goal arm compared to control littermates (Fig. 6f; *t-*test: t_(27)_ = 2.07, *p* = 0.048). When comparing the probe test results for all three time points we found an interaction between genotype and duration of APP_Sw,Ind_ expression (two-way ANOVA: genotype F_(1,58)_ = 0.90, *p* = 0.35; length of APP expression F_(2,58)_ = 2.86, p = 0.065; genotype x length of APP expression F_(2,58)_ = 4.23, *p* = 0.019). Thus, we show here an emerging deficit in spatial reference memory in mature-onset mice and that 12 weeks of mature-onset APP_Sw,Ind_ expression is sufficient to cause a deficit in the Y-water maze task.

### A deficit in basal synaptic transmission at CA3/CA1 synapses is evident in mature-onset APP/tTA mice following 12 weeks of APP_Sw,Ind_ expression

**Fig. 7:**
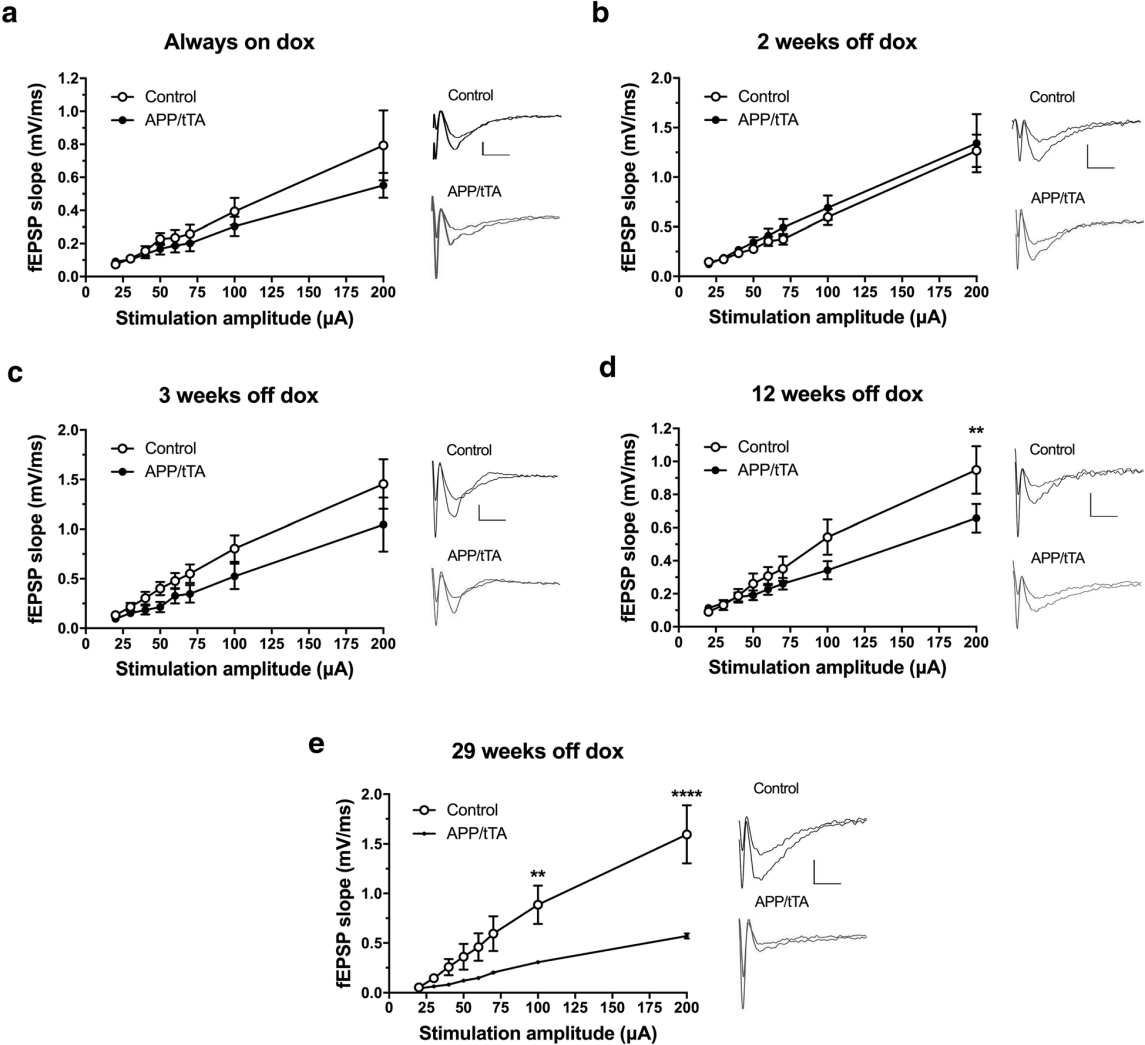
Emergence of a deficit in input-output response in CA3-CA1 synapses. **a** No significant differences between input-output response in APP/tTA mice reared on dox and control littermates (control n = 6, APP/tTA n = 6). **b** Normal input-output response in APP/tTA mice expressing APP_Sw,Ind_ for 2 weeks (control n = 7, APP/tTA n = 7). **c** Normal input-output response in APP/tTA mice expressing APP_Sw,Ind_ for 3 weeks (control n = 8, APP/tTA n = 8). **d** Reduced input-output response in APP/tTA mice expressing APP_Sw,Ind_ for 12 weeks (control n = 11, APP/tTA n = 11). **e** Reduced input-output response in APP/tTA mice expressing APP_Sw,Ind_ for 29 weeks (control n = 6, APP/tTA n = 6). Scale bar calibration: 5 ms, 0.5 mV

We then characterized the emergence of synaptic deficits in mature-onset line 102 mice by performing field recordings at CA3-CA1 synapses in hippocampal slices. I-O curves were used to measure baseline synaptic transmission. Firstly, we analyzed 10–13 week-old mice born and reared continuously on the dox diet (always-on-dox) to assess any baseline effects of transgene leakage. We found that there was no significant effects of genotype in I-O responses between APP/tTA mice and control littermates (Fig. 7a; RM ANOVA: genotype F_(1,10)_ = 0.85, *p* = 0.38; stimulation F_(7,70)_ = 26.44, p < 10^− 4^; genotype x stimulation amplitude F_(7,70)_ = 1.17, *p* = 0.33).

I-O responses were also comparable between control mice and APP/tTA mice expressing the APP_Sw,Ind_ transgene for 2 weeks or 3 weeks with no effect of genotype (2 weeks, Fig. 7b; RM ANOVA: genotype F_(1,12)_ = 0.33, *p* = 0.58; stimulation F_(7,84)_ = 45.88, p < 10^− 4^; genotype x stimulation F_(7,84)_ = 0.16, p = 0.99; 3 weeks, Fig. 7c; RM ANOVA: genotype F_(1,14)_ = 2.03, *p* = 0.18; stimulation F_(7,98)_ = 41.71, p < 10^− 4^; genotype x stimulation F_(7,98)_ = 1.13, p = 0.35). By 12 weeks of APP_Sw,Ind_ expression, however, the I-O responses of APP/tTA mice were significantly reduced compared to littermate controls (Fig. 7d; RM ANOVA: genotype F_(1,20)_ = 1.44, *p* = 0.24; stimulation F_(7,140)_ = 74.82, *p* < 10^− 4^; genotype x stimulation F_(7,140)_ = 4.32, *p* = 0.0002). To further investigate the progressive impairment of basal synaptic transmission, we also analyzed mice expressing APP_Sw,Ind_ for 29 weeks (Fig. 7e) and observed an even bigger reduction in the size of the fEPSPs for a given stimulation intensity, with both an effect of genotype (F_(1,10)_ = 7.70, *p* = 0.02) and an interaction between genotype and stimulation amplitude (F_(7,70)_ = 10.45, p < 10^− 4^).

**Fig. 8:**
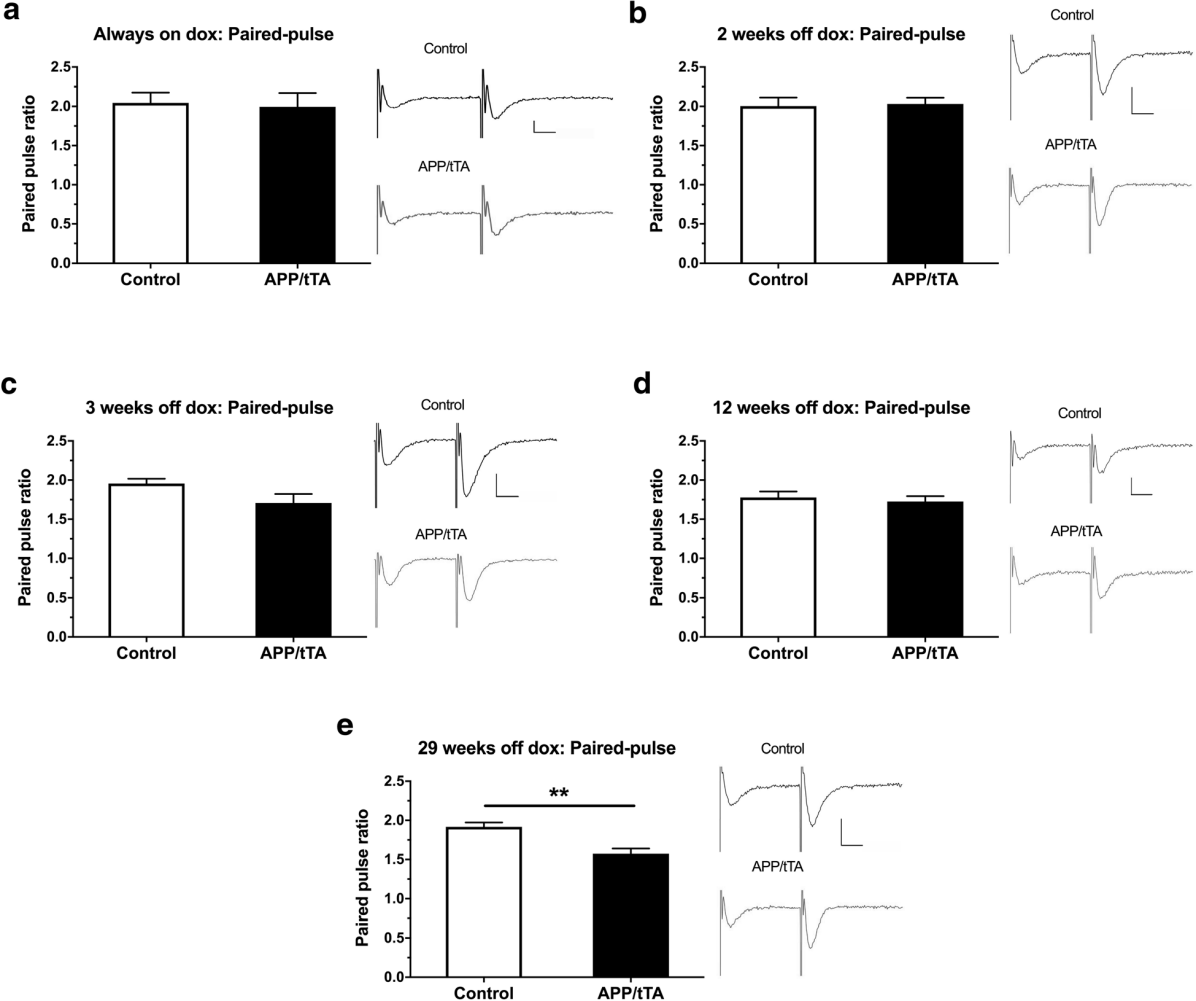
Emergence of a deficit in paired-pulse response in CA3-CA1 synapses. **a** Paired-pulse response was similar in APP/tTA and control mice reared on dox (control mean = 2.1 ± 0.13, n = 6; APP/tTA mean = 2.0 ± 0.18, n = 6). **b** Normal paired-pulse response in APP/tTA mice expressing APP_Sw,Ind_ transgene for 2 weeks (control mean = 2.0 ± 0.11, n = 7; APP/tTA mean = 2.0 ± 0.08, n = 7). **c** Normal paired-pulse response in APP/tTA mice expressing APP_Sw,Ind_ transgene for 3 weeks (control mean = 2.0 ± 0.06, n = 8; APP/tTA mean = 1.7 ± 0.12, n = 8). **d** Normal paired-pulse response in APP/tTA mice expressing APP_Sw,Ind_ for 12 weeks (control mean = 1.8 ± 0.07, n = 11; APP/tTA mean = 1.7 ± 0.07, n = 11). **e** Impaired paired-pulse response in APP/tTA mice expressing APP_Sw,Ind_ for 29 weeks (control mean = 1.9 ± 0.06, n = 6; APP/tTA mean = 1.6 ± 0.07, n = 6). Scale bar calibration: 10 ms, 0.2 mV

We also measured paired pulse facilitation as an indicator of presynaptic function. We found that the PPF response in APP/tTA mice was comparable to control littermates for mice that were always-on-dox (Fig. 8a; *t-*test: t_(10)_ = 0.23, *p* = 0.82) and for those expressing the APP transgene for 2 weeks (Fig. 8b; *t-*test: t_(12)_ = 0.21, *p* = 0.84), 3 weeks (Fig. 8c; *t-*test: t_(14)_ = 1.96, *p* = 0.077), and 12 weeks (Fig. 8d; *t-*test: t_(20)_ = 0.52, *p* = 0.61). In contrast, at the most advanced time point of 29 weeks-off-dox, we did find a significant impairment in PPF responses (Fig. 8e; *t-*test: t_(10)_ = 3.96, *p* = 0.0027). Thus at 29 weeks-off dox we observe a dysregulation of both pre- and post-synaptic function.

### Synaptic plasticity is impaired in mature-onset APP/tTA mice after 3 weeks of APP_Sw,Ind_ expression

**Fig. 9:**
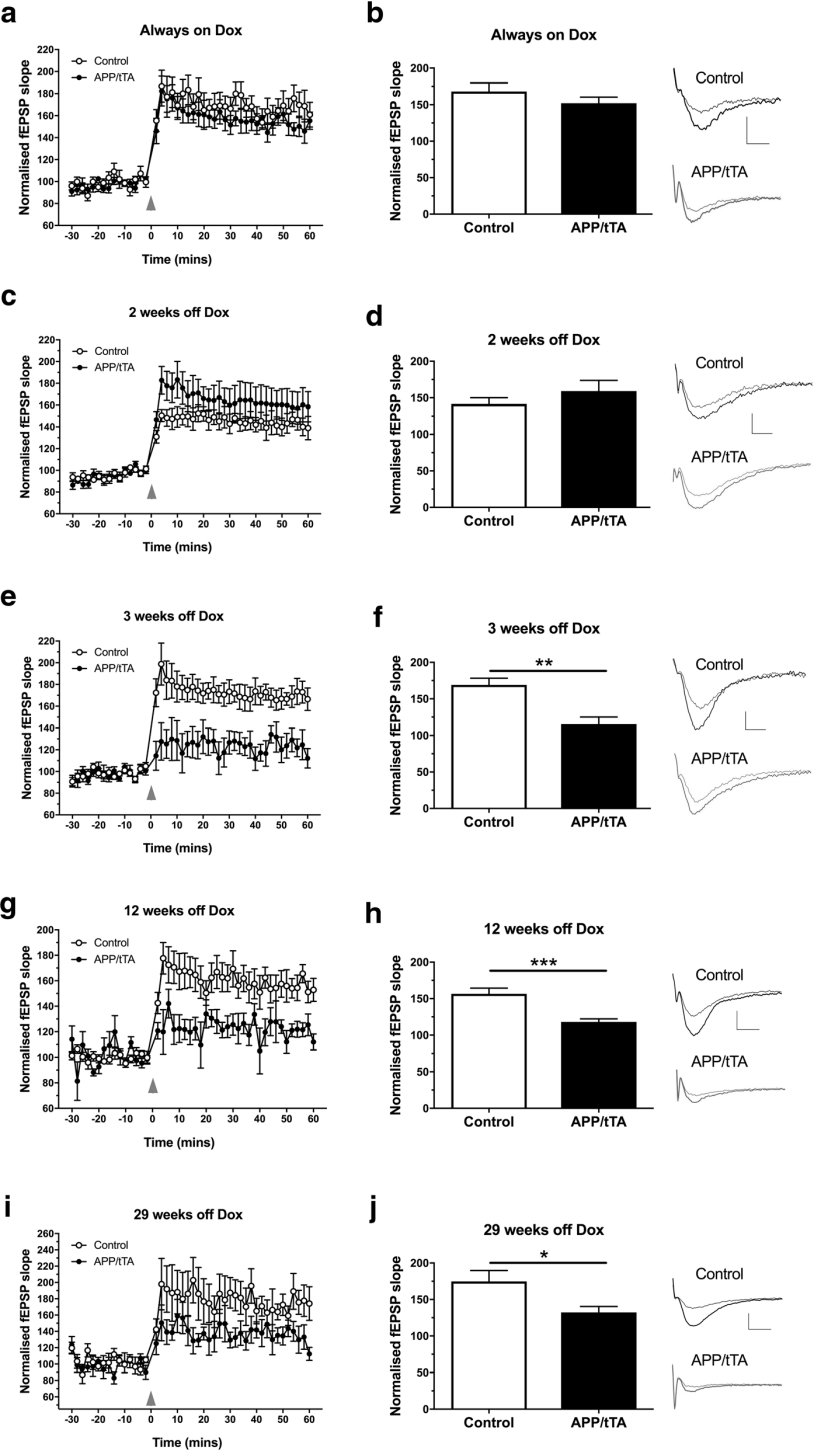
Emergence of a deficit in synaptic plasticity in CA3-CA1 synapses. **a** TBS-induced LTP (arrowhead) was similar between control and APP/tTA mice reared on dox (always-on-dox). **b** End LTP value, averaged 50–60 min after TBS, showed that APP/tTA mice reared on dox exhibited a similar level of LTP to control littermates (control mean = 168.1 ± 11.74, n = 6; APP/tTA mean = 152.1 ± 8.34, n = 6). Example traces are shown for time points immediately before (thin line) and 60 min after (thick line) LTP induction. **c** TBS-induced LTP was normal in APP/tTA mice expressing APP_Sw,Ind_ for 2 weeks (2 weeks-off-dox). **d** APP/tTA mice expressing APP_Sw,Ind_ for 2 weeks exhibited a similar LTP response as control littermates (control mean = 141.2 ± 8.46, n = 6; APP/tTA mean = 159.0 ± 14.66, n = 6). **e** TBS-induced LTP was impaired in APP/tTA mice expressing APP_Sw,Ind_ for 3 weeks (3 weeks-off-dox). **f** End LTP response was lower in APP/tTA expressing APP_Sw,Ind_ for 3 weeks (control mean = 169.1 ± 9.09, n = 8; APP/tTA mean = 115.4 ± 9.69, n = 6). **g** TBS-induced LTP was impaired in APP/tTA mice expressing APP_Sw,Ind_ for 12 weeks (12 weeks-off-dox). **h** End LTP response was lower in APP/tTA expressing APP_Sw,Ind_ for 12 weeks (control mean = 156.5 ± 7.75, n = 9; APP/tTA mean = 118.0 ± 4.17, n = 9). **i** TBS-induced LTP was reduced in APP/tTA mice expressing APP_Sw,Ind_ for 29 weeks (29 weeks-off-dox). **j** End LTP response was lower in APP/tTA expressing APP_Sw,Ind_ for 29 weeks (control mean = 174.7 ± 15.06, n = 6; APP/tTA mean = 132.2 ± 8.24, n = 6). Labels in a apply to all LTP plots in figure. Scale bar calibration: 5 ms, 0.5 mV

We also assessed synaptic plasticity in the CA3-CA1 pathway and found that the amount of LTP induced was similar between APP/tTA and control mice born and raised continually on the dox diet (Fig. 9a-b; *t-*test: t_(10)_ = 1.11, *p* = 0.29), indicating no effect of transgene leakage. LTP levels were also comparable between mature-onset APP/tTA mice expressing APP_Sw,Ind_ for 2 weeks and control littermates (Fig. 9c-d; *t-*test: t_(10)_ = 1.03, p = 0.33), despite the substantial increase in APP, and Aβ levels (Fig. 3). In marked contrast, however, following 3 weeks of APP_Sw,Ind_ expression, we then observed a substantial reduction in LTP in mature-onset APP/tTA mice compared to control littermates (Fig. 9e-f; *t-*test: t_(12)_ = 3.99, *p* = 0.0018). Impaired LTP was also observed in mature-onset APP/tTA mice expressing the APP_Sw,Ind_ transgene for 12 weeks-off-dox (Fig. 9g-h; *t-*test: t_(16)_ = 4.37, *p* = 0.0005) and 29 weeks-off-dox (Fig. 9i-j; *t-*test: t_(10)_ = 2.47, *p* = 0.033).

Thus, we show that 3 weeks of APP_Sw,Ind_ expression is sufficient to drive a significant deficit in synaptic plasticity in the mature-onset line 102 model, which coincides with the emergence of a spatial working memory deficit (Fig. 4c). Interestingly, this deficit precedes the significant impairment in basal synaptic transmission and spatial reference memory that were observed at 12 weeks-off-dox (Fig. 7). Notably, there were no significant differences between the magnitude of LTP deficit in mature-onset APP/tTA mice between 3 and 12 weeks (*t-*test: t_(13)_ = 0.29, *p* = 0.78), 3 and 29 weeks (*t-*test: t_(10)_ = 1.35, *p* = 0.21) and 12 and 29 weeks (*t-*test: t_(13)_ = 1.69, *p* = 0.11), indicating that the maximum LTP impairment is evident from 3 weeks-off-dox.

### Early deficit in synaptic plasticity can be reversed by suppressing APP_Sw,Ind_ expression

**Fig. 10:**
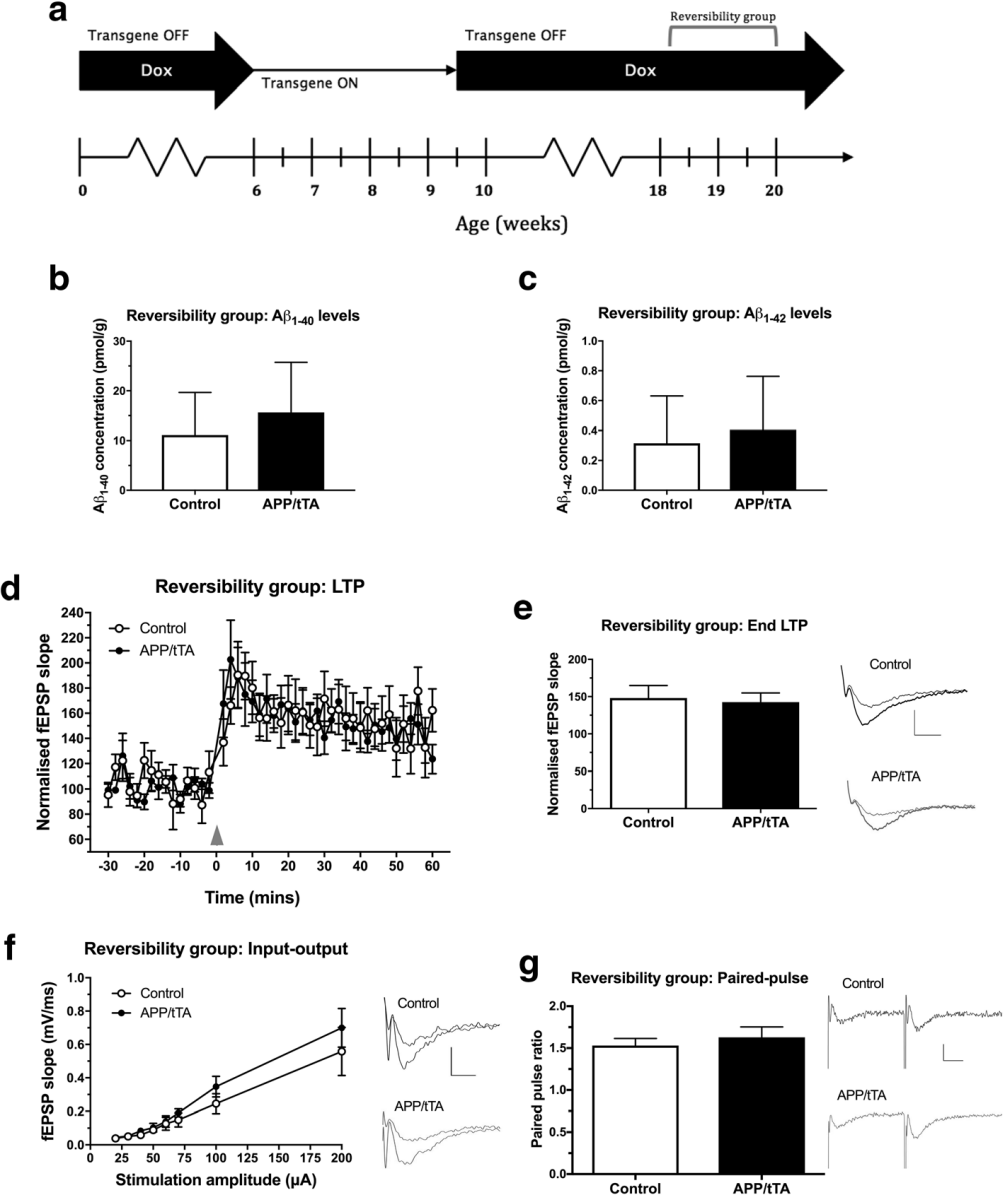
Suppressing APP expression and Aβ levels allows recovery of LTP deficit in APP/tTA mice. **a** Experiment timeline; mice allocated to the reversibility group expressed APP_Sw,Ind_ for 3.5 weeks before being put back on the dox diet for 9–11 weeks to suppress further APP_Sw,Ind_ expression. **b** Aβ_40_ levels in hippocampal lysates of APP/tTA mice from the reversibility group were similar to control genotype levels. In comparison, shaded area shows levels of Aβ_40_ for age-matched APP/tTA mice from the 12 weeks-off-dox group (12 weeks-off-dox: control n = 9, APP/tTA n = 7; Reversibility study: control n = 6, APP/tTA n = 6). **c** Aβ_42_ levels in hippocampal lysates of APP/tTA mice from the reversibility group returned to control levels. Shaded area shows levels of Aβ_42_ for APP/tTA mice from the age-matched 12 weeks-off-dox group (12 weeks-off-dox: control n = 9, APP/tTA n = 7; Reversibility study: control n = 6, APP/tTA n = 6). **d** TBS-induced LTP (arrowhead) was similar between control and APP/tTA mice from the reversibility study group. **e** End LTP values, averaged 50–60 min after TBS, were similar between control and APP/tTA mice from the reversibility study group (control mean = 148.0 ± 16.86, n = 7; APP/tTA mean = 142.6 ± 12.24, n = 7). LTP trace shown for time points immediately before (thin line) and 60 min after (thick line) LTP induction. Scale bar calibration for LTP trace: 5 ms, 0.5 mV. **f** A similar input-output response was observed in the CA3-CA1 pathway of control and APP/tTA mice from the reversibility study (control n = 8, APP/tTA n = 8). Scale bar calibration for input-output trace: 5 ms, 0.5 mV. **g** Paired-pulse response was similar between control and APP/tTA mice from the reversibility group (control mean = 1.5 ± 0.08, n = 8; APP/tTA mean = 1.6 ± 0.12, n = 8). Scale bar calibration for paired-pulse traces: 10 ms, 0.2 mV

Previous work has shown that Aβ disrupts synaptic protein scaffolds by activating intracellular pathways that promote synapse disassembly: observed as a reduction in PSD thickness [49] and underpinned by a dispersal of PSD components [44] prior to neurodegeneration. We hypothesised that the earliest deficit in synaptic plasticity observed in mature-onset APP/tTA mice (Fig. 9e-f) after 3 weeks of Aβ accumulation expression might be reversible, and that switching off APP_Sw,Ind_ expression would be sufficient to restore synaptic plasticity. Mice were therefore taken off dox at six weeks of age and allowed to express the APP_Sw,Ind_ transgene for 3.5 weeks. Following this, mice were put back on the dox diet for 9–11 weeks before being tested at 18–20 weeks of age (Fig. 10a, “Reversibility group” on the experimental timeline).

We assessed total levels of Aβ_40_ and Aβ_42_ in hippocampal lysates of APP/tTA mice from the reversibility group and control littermates (Fig. 10b-c), and compared these against age-matched mature-onset mice that expressed the APP_Sw,Ind_ transgene for 12 weeks (Fig. 3d-e, 12 weeks-off-dox). As expected, putting APP/tTA mice back on the dox diet caused a significant reduction in Aβ_40_ (Fig. 10b; two way ANOVA: genotype F_(1,24)_ = 23.72, *p* < 10^− 4^; dox diet F_(1,24)_ = 16.69, *p* = 0.0004; genotype x dox diet F_(1,24)_ = 21.21, *p* = 0.0001) and Aβ_42_ (Fig. 10c; two way ANOVA: genotype F_(1,24)_ = 33.14, p < 10^− 4^; dox diet F_(1,24)_ = 35.41, p < 10^− 4^; genotype x dox diet F_(1,24)_ = 32.83, p < 10^− 4^), back to the levels found in control littermates.

We next sought to assess if this reduction in Aβ levels was sufficient to rescue the observed deficit in synaptic plasticity seen in mature-onset APP/tTA mice following 3 weeks of APP_Sw,Ind_ expression (Fig. 10d-e). Following TBS, both APP/tTA mice from the reversibility group and control littermates in interleaved experiments exhibited similar LTP (Fig. 10d-e; *t-*test: t_(12)_ = 0.26, *p* = 0.80). Thus, the early deficit in synaptic plasticity observed following 3 weeks of APP_Sw,Ind_ expression (Fig. 9e-f) is reversible. Importantly, inhibiting further APP expression and Aβ accumulation also prevented the progressive reduction in baseline synaptic transmission and I-O deficit observed in mature-onset mice expressing APP_Sw,Ind_ for 12 weeks. APP/tTA mice from the reversibility group also exhibited normal I-O (Fig. 10f; RM ANOVA: genotype F_(1,14)_ = 0.77, *p* = 0.40; stimulation F_(7,98)_ = 37.92, *p* < 10^− 4^; genotype x stimulation F_(7,98)_ = 0.64, *p* = 0.72) and PPF responses (Fig. 10g; *t-*test: t_(14)_ = 0.65, *p* = 0.53) compared to control littermates.

Thus, our mature-onset data show that in APP/tTA mice, synaptic and cognitive deficits appear progressively: synaptic plasticity and spatial working memory are first affected, and then impairment of basal synaptic transmission and spatial reference memory emerge later. Unlike mature-onset APP/tTA mice expressing APP for 12 weeks, developmental onset APP/tTA mice expressing APP for 12 weeks show normal spatial reference memory in the Y-maze escape task and normal CA3-CA1 synaptic plasticity, despite showing deficits in basal synaptic transmission and working memory. Our results demonstrate that despite expressing the APP_Sw,Ind_ transgene for a similar length of time, there are key and substantial differences between mature-onset and developmental onset expression.

## Discussion

Although the precise time of onset of AD is ill-defined, it is increasingly recognized that understanding the early events in disease progression may lead to more effective therapeutic interventions [24, 28, 58]. Here we have used the inducible line 102 model to map the emergence of Aβ-mediated cognitive and synaptic deficits [23, 71]. We firstly addressed the differences between developmental-onset and mature-onset overexpression of APP and Aβ. We found that although there were similar levels of APP expression in both models expressing APP_Sw,Ind_ for 12 weeks, it was not possible to match exactly the Aβ expression levels in developmental-onset and mature-onset mice; Aβ_40_ levels were seven fold and Aβ_42_ levels were twenty fold higher in developmental-onset mice. These results add support to the evidence for differences in APP processing mechanisms between the neonatal and adult brain [8]. Importantly, the inducible line 102 model provided a gradual increase of Aβ at physiologically relevant concentrations which are within the pM range observed in postmortem AD brain tissue [39, 40].

Developmental-onset mice showed a substantial disruption of basal synaptic transmission consistent with previous studies in several APP lines [16, 31, 33]. Nevertheless, similar to other young-adult developmental APP models, we found that despite high levels of Aβ, developmental onset APP/tTA mice exhibited normal levels of NMDAR-dependent hippocampal LTP in CA3-CA1 synapses (which was inhibited by APV, data not shown) [13, 19, 26, 27, 32, 42]. These results are in stark contrast to the impaired LTP we observed in mature-onset mice with a similar duration of APP expression, and even though there were lower Aβ levels in the mature-onset model. Indeed, a stronger inhibition of LTP would be expected from a higher dose of Aβ in vivo present in the developmental-onset mice [25]. These results suggest either compensation or reduced vulnerability to expression of APP_Sw,Ind_ and Aβ in young brains. Previous research shows that there are important age-dependent differences in signal transduction cascades for LTP [70] which can render hippocampal LTP insensitive to acute Aβ application in postnatal mice [62]. The highly plastic brains of developing mice (Daw et al. 2008) may be able to prevent the immediate effects of Aβ and may be able to adapt their plasticity mechanisms to long term exposure, as shown by our normal LTP results in 12 week developmental onset mice. However, it is clear that basal synaptic transmission is still impaired in developmental-onset animals (Fig. 2c-d). Conversely, it is possible that once this period of developmental plasticity has passed there is a decreasing capacity for the brain to functionally compensate for damage caused by Aβ as demonstrated by the persistent loss of LTP in our mature-onset model up to 29 weeks-off-dox. Further research will be required to test whether the synapse loss mechanisms in developmental-onset vs mature-onset mice are the same and whether the differences in these reflect the mechanisms of synapse loss in familial and sporadic AD, respectively.

Developmental onset line 102 mice (12 weeks of age) performed poorly in the MWM (Fig. 1b-d). Their acquisition and probe trial readouts were highly impaired. They performed at chance levels, similar to other developmental APP models [48, 69]. However, on further inspection of the data we observed a highly thigmotaxic phenotype which could contribute to their MWM deficit. This was also evident in mice with 2-weeks-off-dox expression. It was thus not possible to determine whether there was a genuine learning impairment in the open field watermaze paradigm, independent of the effects of thigmotaxis. However, when tested with an alternative hippocampal-dependent, spatial reference memory task which is unaffected by thigmotaxis (Y-water maze), APP/tTA mice acquired the task as well as control littermates. Taken together, these behavioural results highlight the potential influence that levels of thigmotaxis can have on water maze acquisition and performance [43].

Our results suggest that the residual hippocampal synaptic function and plasticity in developmental-onset mice is able to support performance in a long-term memory task in the Y-Water maze. Moreover, despite normal levels of LTP in developmental onset APP mice, we observed highly impaired CA3-CA1 basal synaptic transmission. This deficit could underpin the impaired performance in alternation in the T-maze given the high sensitivity of this task to hippocampal dysfunction [4]. In contrast to developmental onset mice, our analysis of mature-onset APP expressing mice, shows that that hippocampal synaptic plasticity is greatly diminished following 3 weeks of APP_Sw,Ind_ expression whereas basal synaptic transmission is not affected until later –following 12–29 weeks of APP_Sw,Ind_ expression. The sequence of events we have described here, suggests that a lack of LTP in the mature-onset model would potentially lead to lower synaptic transmission over time. The decrease in paired pulse facilitation we observe, both in the developmental model and at 29-weeks-off-dox, may reflect a compensatory effect of enhanced presynaptic release probability as a result of reduced basal transmission.

Notably, the loss of plasticity we observed at 3-weeks-off-dox shares some similarities with results from acute application of nanomolar Aβ concentrations onto acute hippocampal slices which blocked LTP, without affecting basal synaptic transmission [56, 63, 65]. However, the normal plasticity we observed at 2-weeks-off-dox after a gradual increase of Aβ to pM levels is at odds with the acute effect of Aβ within hours of a step change to nM concentrations. The gradual increase of Aβ reaching chronic pM concentrations may allow for early compensation given the capacity of neural circuits for physiological adaptations such as homeostatic plasticity mechanisms [46, 59] which may differ in developmental-onset and mature-onset animals. Further analysis will be required to test whether these compensatory mechanisms are at play and their relevance to human synapses when CSF Aβ levels change prior to and during plaque deposition in AD [9, 61]. Moreover, it would be of interest to analyse whether the age of onset of APP expression in line 102 mice (e.g. mature-onset vs ageing-onset) can have an impact on the emergence and progression of cognitive and synaptic impairment.

We observed a dissociation between CA3-CA1 LTP levels and spatial reference memory performance in mature-onset animals. Mice with 3 weeks of mature-onset APP expression, exhibited normal Y-water maze acquisition and performance during the subsequent probe trial, despite impaired LTP. Although the idea that activity-dependent modification of synaptic strength provides a neural substrate for learning and memory has been intensively investigated for many years [10, 37], the precise relationships between different forms of synaptic plasticity and different aspects of memory performance remain to be fully resolved [3]. Nevertheless, NMDAR dependent synaptic plasticity plays an important role in short term memory processes that may contribute to spatial working memory performance [3, 50] and the 3 weeks-off-dox mice exhibited a spatial working memory deficit in spontaneous alternation in the T-maze which proved the more sensitive measure of the emergent cognitive decline [4, 50]. Notably, working memory deficits are a key feature of early Alzheimer’s disease –whereby impairment in the “registration, storage, and retrieval of new information” that impairs daily living is essential for diagnosis [34], while long term memory is more associated with advanced AD stages. This suggests that line 102 is a suitable mouse model for the emergence of AD that could guide further mechanistic analyses and therapeutic testing.

A number of strategies are being pursued to lower Aβ levels and reverse their effects in the brain [23, 54, 67]. We show here that by increasing and then subsequently decreasing APP expression and de novo Aβ production, we were able to reverse the early deficits in LTP we had observed previously. Interestingly, we found that reversing the LTP deficit also prevented the subsequent reduction in basal synaptic transmission (Fig. 10d-g). Although our experiments do not determine whether loss of plasticity and synapse loss in the mature-onset model are causally related, our data suggest that the loss of LTP could be either a precursor or driver of the subsequent loss of synaptic input manifested as a reduction in I-O curves. Thus, our findings suggest that loss of synaptic plasticity is a very early event that could ultimately underpin both short and long memory loss in the line 102 mature-onset model, and therefore understanding the mechanisms of LTP impairment in this model could guide us in the search for therapeutic targets in early AD.

Although very little is known regarding the loss of plasticity in the human brain in AD, one study [5] found that plasticity following paired associative stimulation is already impaired in individuals with mild to moderate AD. This suggests that the loss of synaptic plasticity we observe in mature-onset line 102 mice is relevant to the early phase of disease and given its reversibility, that it may be amenable to pharmacological intervention. It will be of interest to discover if and when these synaptic deficits are no longer reversible in mice, and then in humans [64] in order to time neuroprotective interventions accordingly. Thus, our work provides a platform to further dissect the cellular mechanisms that underpin early cognitive deficits and loss of plasticity in early AD [60].
